# Risperidone regulates the expression of schizophrenia-related genes in the forebrain of adult male mice

**DOI:** 10.3389/fnmol.2026.1844705

**Published:** 2026-05-29

**Authors:** Magdalena Ziemiańska, Mateusz Zięba, Anna Radlicka-Borysewska, Łukasz Szumiec, Sławomir Gołda, Małgorzata Borczyk, Marcin Piechota, Michał Korostyński, Jan Rodriguez Parkitna

**Affiliations:** 1 Department of Molecular Neuropharmacology, Maj Institute of Pharmacology, Polish Academy of Sciences, Krakow, Poland; 2 Laboratory of Pharmacogenomics, Maj Institute of Pharmacology, Polish Academy of Sciences, Kraków, Poland

**Keywords:** antipsychotics, forebrain, gene expression, mouse, risperidone, schizophrenia, spatial transcriptomics

## Abstract

**Introduction:**

Risperidone is a widely used antipsychotic that reduces psychotic symptoms through modulation of monoaminergic signaling. At the cellular level, however, its effects extend beyond receptor antagonism and induce spatially discrete transcriptional responses across forebrain structures, particularly in the basal ganglia and frontal cortex.

**Methods:**

We applied sequencing-based spatial transcriptomics to the forebrain of male C57BL/6N mice following acute risperidone treatment (0.5 mg/kg, i.p.). Unsupervised clustering of spatial transcriptomic profiles accurately delineated cortical divisions, cortical layers, and basal ganglia subregions. Differential expression analysis within anatomically defined clusters, performed using a customized statistical framework, revealed transcriptional patterns that were highly structure-specific and distinct between cortical and basal ganglia regions.

**Results:**

Acute risperidone treatment significantly affected 95 transcripts across 12 brain regions. The most prominent transcriptional changes were concentrated in ventral forebrain structures, including the olfactory tubercle (25 differentially expressed transcripts), diagonal band nucleus (22), corpus callosum and commissures (13), and lateral septal nucleus (9). Importantly, 18 of these 95 genes have previously been implicated in schizophrenia, including *Olig2*, *Smpd3*, and *Cacna1i*.

**Discussion:**

Together, these findings indicate that acute treatment with risperidone in male mice exerts pronounced molecular effects in medial and ventral forebrain regions with high oligodendrocyte and glial cell abundance. Moreover, enrichment analysis points to a molecular convergence between risperidone-induced transcription and genetic pathways implicated in schizophrenia.

## 1 Introduction

Antipsychotic drugs act on monoaminergic signaling to alleviate the positive symptoms of schizophrenia. While they exert their effects primarily through dopamine D2 receptor blockade, second-generation antipsychotics are also potent antagonists of serotonin 5-HT2A receptors and interact with various monoaminergic and cholinergic targets (Seeman, 2002; Miyamoto et al., 2005; Meltzer, 2013). A high relative ratio of 5-HT2A and D2 receptor antagonism defines atypical antipsychotics, which cause fewer adverse extrapyramidal effects while still effectively reducing psychosis (Meltzer, 2013). It is important to note that the dopaminergic mechanism of action of antipsychotic drugs is a major piece of evidence supporting the dopamine hypothesis of schizophrenia’s etiology (Snyder et al., 1974; Howes et al., 2015, 2017). Nonetheless, despite progress in psychosis pharmacotherapy, the root causes of schizophrenia remain unclear, treatments are limited in addressing negative symptoms, and ongoing research into how antipsychotics work continues to reveal increasing complexity.

A key approach to unraveling these molecular mechanisms is to profile drug effects on gene expression (Robertson et al., 1994; Harrison and Weinberger, 2005; Nakamura and Takata, 2023). Changes in transcription support long-term plasticity, so drug-induced gene expression profiling offers insight into neuronal mechanisms behind both immediate and lasting effects of psychotropic drugs (McClung and Nestler, 2008; Zygmunt et al., 2019). A single acute dose of an antipsychotic drug is sufficient to robustly induce activity-regulated transcripts, especially immediate-early genes (Dragunow et al., 1990; Robertson and Fibiger, 1992; Korostynski et al., 2013; De Bartolomeis et al., 2017). The distribution of drug-induced transcriptional changes varies by region, with the nucleus accumbens and frontal cortex most affected, and differences between drugs in regional expression patterns (De Bartolomeis et al., 2017). It has been observed that typical antipsychotics exert stronger effects on activity-regulated genes in the dorsal striatum. In contrast, atypical antipsychotics, which tend to cause fewer extrapyramidal adverse effects, show relatively greater effects in the prefrontal cortex and nucleus accumbens (Robertson et al., 1994; Wan et al., 1995; Deutch and Duman, 1996). At the same time, dopaminergic dysfunction in schizophrenia also exhibits a distinct spatial pattern, with hyperactivity in the basal ganglia and hypoactivity in the prefrontal cortex being the prevailing view (Tzschentke, 2001; Guillin et al., 2007). Therefore, the spatial pattern of antipsychotic effects correlates with their effectiveness and may predict the severity of extrapyramidal side effects.

Early studies on transcriptional responses to psychotropic drugs concentrated on selected immediate-early genes such as *Fos*. The use of microarrays (Fehér et al., 2005; Ikeda et al., 2010; Korostynski et al., 2013) and later RNA-sequencing technologies (Crespo-Facorro et al., 2015; Zygmunt et al., 2018) expanded this approach to transcriptome-wide analyses, allowing unbiased detection of drug effects on gene expression. These studies identified specific cellular and molecular pathways involved in pharmacological treatments, such as the neuroplasticity-related MAPK signaling pathway (Korostynski et al., 2013) or calcium signaling (Fehér et al., 2005). Notably, these approaches typically focus on individual brain regions or specific neuronal populations due to technical limitations and cannot assess the broader spatial organization of transcriptional responses across the brain. A prime example is the reported similarity in transcriptomic responses after treatment with both antipsychotics and psychostimulants (Korostynski et al., 2013; Zygmunt et al., 2019). Although these changes occur in different cell populations (Bertran-Gonzalez et al., 2008), they become indistinguishable in the homogenized tissue. Furthermore, most psychotropic drugs activate glucocorticoid receptor-dependent transcription in glial cells, probably reflecting broad adaptations to altered neuronal activity (Zygmunt et al., 2019). Recently, the first single-cell RNA-seq analysis of gene expression induced by antipsychotics in the striatum identified highly drug-specific gene patterns and revealed transcriptional changes in microglia (Abrantes et al., 2022). Therefore, while single-cell analysis offers unprecedented insight into the molecular effects of antipsychotics, the method remains limited to specific brain areas due to technical constraints.

Here, we perform a spatial analysis of acute risperidone-induced gene expression changes in the murine forebrain, providing a baseline characterization of antipsychotic effects within the brain’s native tissue architecture (Ståhl et al., 2016). We focused on risperidone, as it is widely used in the treatment of psychoses, schizophrenia, and bipolar disorder (Chopko and Lindsley, 2018) and has been shown to have robust effects on gene expression (Fehér et al., 2005; Korostynski et al., 2013; Zygmunt et al., 2018). Our previous time-course study indicated that the 2-h post-treatment time point yields pronounced, distinct gene expression changes following acute antipsychotic treatment (Korostynski et al., 2013). Building on these findings, the present study focuses on region-specific transcriptional profiling at this peak time point, thereby providing a spatial complement to earlier temporal analysis. Our results demonstrate that risperidone induces spatially discrete transcriptional changes that affect the expression of genes previously implicated in schizophrenia genetics.

## 2 Methods

### 2.1 Animals

Experiments were performed on adult male mice (10-week-old C57BL/6 N, Charles River Laboratories, Germany) weighing 20–26 g. Mice were housed 4 per cage at the animal facility of the Maj Institute of Pharmacology of the Polish Academy of Sciences under standard conditions (22 °C, 12/12 h light/dark cycle) with free access to food and water. All animal experiments were planned following the ARRIVE guidelines (Percie du Sert et al., 2020), and performed following the European and Polish law (Directive 2010/63/UE, European Convention for the Protection of Vertebrate Animals Used for Experimental and other Scientific Purposes ETS No.123, and Polish Law Dz.U. 2015 poz. 266). All procedures were approved by the II Local Institutional Animal Care and Use Committee in Kraków (permit no. 136/2022). The total number of animals used in the experiments was 12. Animals were randomly assigned to saline or risperidone treatment groups. Sample sizes were based on our prior work on the spatial analysis of levodopa effects (Radlicka-Borysewska et al., 2025).

### 2.2 Drug treatment and tissue preparation

Risperidone (cat. no. 2865, Tocris, Biotechne, UK) was suspended in a 1% v/v Tween 80 solution (cat. no P1754, Merck, USA) in saline by sonication. Mice were habituated to intraperitoneal injections with saline for two consecutive days before receiving a single acute injection of risperidone (0.5 mg/kg in a volume of 5 μL/g body weight) or vehicle. The dose is based on previous reports showing that doses up to 1 mg/kg produced no attenuation in the startle response or pre-pulse inhibition (Le Pen and Moreau, 2002; Baba et al., 2019). Animals were killed 2 h after risperidone treatment by cervical dislocation, as approved by the Animal Care and Use Committee. Brains were collected, rinsed with cooled saline (4 °C), and coated in OCT compound (cat. no. KMA010000A, Cell Path, UK). Then, they were snap-frozen in Peel-A-Way® embedding molds (cat no. E6032, Polysciences, USA) by immersion in isopentane chilled with dry ice (−78 °C). Samples were stored at −80 °C in sealed containers for long-term preservation. Before slicing, brains embedded in the OCT compound were equilibrated to the cryostat chamber temperature (−20 °C) for at least 30 min. Tissue blocks were trimmed, and 10 μm-thick coronal sections were cut using a CM 3050S cryostat microtome (RRID:SCR_020214, Leica Microsystems, Germany) at an object temperature of −13 °C and a chamber temperature of −20 °C. For spatial transcriptomics, one coronal section containing the prefrontal cortex and rostral striatum (1.54–1.18 mm from bregma) was collected from each animal. Coronal sections were mounted one per capture area on a pre-cooled (−20 °C) Visium Spatial slide (RRID:SCR_023571, 10x Genomics, USA). Sections were fixed in methanol at −20 °C for 30 min and stained with hematoxylin and eosin according to the manufacturer’s protocol (Tissue Preparation Guide Rev. D; Methanol Fixation, H&E Staining & Imaging for Visium Spatial Protocol Rev. D, Visium Spatial Protocols). Imaging was performed using a Leica DMi8 microscope with Leica Application Suite X v3.5.2.18963 software. Each section was imaged in bright-field mode using an HC PL FLUOTAR 10×/0.30 NA (dry) objective and a DFC 7000 camera with the following acquisition settings: 8-bit color depth, gain 10, exposure time 4.5 ms, and a resolution of 1920 × 1,440 pixels. The images were saved as .tif files and used to determine the surface area of the array covered by tissue in Loupe Browser v6.0 (RRID:SCR_018555, 10x Genomics). Processed images were then used to generate .json files for further analysis in the spaceranger-MACS3-Nanopore-spaceranger processing pipeline.

### 2.3 Visium libraries preparation and sequencing

After imaging, brain slices were enzymatically permeabilized for 8 min, followed by incubation in reverse transcription master mix at 53 °C for 45 min. The resulting cDNA was incubated with 0.08 M KOH (cat no. P4494, Merck, USA) and washed with EB buffer (cat no. 19086, Qiagen, Germany). Next, second-strand synthesis (65 °C, 15 min), denaturation (0.08 M KOH, 10 min), and cDNA amplification were performed. The total number of cDNA amplification cycles was determined by qPCR using KAPA SYBR FAST qPCR Master Mix (cat no. KK4600, Roche, Switzerland) with 1 μL of cDNA solution. Amplified cDNA was purified using SPRIselect beads (cat. no. B23318, Beckman Coulter, USA). Quantification and quality control were conducted using Bioanalyzer 2,100 (RRID:SCR_019715, Agilent, Palo Alto, CA, USA) with High Sensitivity DNA Kit reagents (cat. no. 5067–4,626, Agilent, Palo Alto, CA, USA) to determine total cDNA yield. Spatial gene expression libraries were constructed according to the manufacturer’s guidelines (RRID:SCR_023571, Visium Spatial Gene Expression Reagent Kits User Guide Rev. G and Visium Spatial Gene Expression Library Construction). First, cDNA samples were fragmented with post-fragmentation end-repair and A-tailing (32 °C for 5 min, 65 °C for 30 s). Next, double-sided size selection with SPRIselect was done before the adaptor ligation and post-ligation cleanup. cDNA amplification with a unique index pair was conducted according to the protocol, followed by a second double-sided size selection with SPRIselect beads. The quality of the libraries was assessed using the Bioanalyzer 2,100 with High Sensitivity DNA Kit reagents. Samples were stored at −20 °C for long-term preservation. Pooling and sequencing of the libraries were performed externally at CeGaT (Germany) using the NovaSeq 6,000 Sequencing System (RRID:SCR_016387, Illumina, SP flow cell). Paired-end, dual-indexed RNA sequencing was performed using recommended parameters for Visium Spatial Gene Expression - read 1, 28 cycles; i7 index, 10 cycles; i5 index, 10 cycles; and read 2, 50 cycles, 200 million read pairs per sample.

### 2.4 RNA sequencing data processing

RNA sequencing yielded on average ~332 million total reads per library. Raw RNA-seq data quality was assessed with FastQC (RRID:SCR_014583, version 0.11.5-cegat). Samples had an average Q30 score of 92.95%. The data were processed using the Space Ranger v1.3.1 analysis pipelines (RRID:SCR_025848, https://www.10xgenomics.com/support/software/space-ranger/latest). The reference genome, tissue section micrographs, and FASTQ files were used to detect tissue boundaries, identify fiducial frame markers, and perform barcode/UMI counting. Reads were aligned to the mm10 mouse reference genome (GRCm38/mm10; 2020-A; June 23, 2020). Analysis of the dataset showed that a fraction of reads mapped outside canonical transcript boundaries defined in the reference GTF. To account for this, we implemented a data-driven annotation pipeline to extend UMI quantification beyond standard exonic windows.

### 2.5 Peak processing, validation, and custom reference generation

The resulting BAM alignment files were merged into a single experiment-wide file using Samtools (RRID:SCR_002105, https://github.com/samtools/samtools). MACS3 v3^1^ was applied to the merged BAM files to identify genomic regions with enriched read coverage independent of reference transcript annotations. Strand orientation was determined using Samtools 1.17 based on coverage depth on both strands. Peaks were filtered based on the following criteria: amplitude > 400, read counts per peak > 1,200, count-to-amplitude ratio > 1.4, score > 350, and summit −log₁₀(p) > 35. The resulting peaks were then annotated to genes or long terminal repeat sequences using a one-sided margin of 30,000 bp from the gene boundary. To refine these annotations, the coordinates were cross-referenced with long-read sequencing data from a previous mouse striatum study (Chrószcz et al., 2025), and only peaks located within a ± 30,000 bp window from the 5′ end of the transcript were retained. A total of 15,243 supported peaks corresponding to 10,493 protein-coding genes were converted into a custom gene transfer format file and used to generate a new reference transcriptome with the Spaceranger-mkref pipeline.^2^ The resulting custom reference and FASTA files were then processed with the Spaceranger-count pipeline to generate feature-barcode matrices quantifying transcript abundance at each tissue spot.

### 2.6 Cluster identification and marker validation

After alignment to the reference genome, highly variable genes were identified in Seurat v4.4.0 using the variance-stabilizing transformation method, with the top 2,000 features retained and then z-score transformed. Principal component analysis was performed on these selected genes, and the first 30 principal components were used to construct a shared nearest-neighbor graph. Clusters were identified at a resolution of 0.4, and UMAP visualization was generated from the same dimensional reduction space. Gene markers for each cluster were defined as genes with significantly higher expression in that cluster than in all other spots, as determined by the Mann–Whitney U test. Markers were required to have a minimum log₂ fold change of 0.5 and to be detected in at least 25% of spots within the cluster. The identification of structure- and tissue-specific markers was validated using marker combinations reported in the literature and relevant databases (see Supplementary Table 1).

### 2.7 Differential gene expression analysis

For downstream statistical analyses, quantile normalization was applied within each cluster, and average transcript expression was evaluated using a customized bioinformatics workflow. Clusters containing fewer than 20 spots were excluded from further analysis, corresponding to 1.5% of clusters in the risperidone dataset. For each remaining cluster, mean relative expression values were calculated per sample, yielding one cluster-level mean expression value per animal, and compared between treatment groups (saline vs. risperidone) using Mann–Whitney U tests. Differential gene expression was considered significant at *p* < 0.01, |log₂ fold change| ≥ 0.8, and a minimum mean transcript abundance of 0.2 in at least one group. The false discovery rate (FDR) was estimated separately for each cluster using Monte Carlo permutation tests based on random permutation of sample assignments across the 12 samples for 1,000 iterations, with the clustering resolution fixed at 0.4. Empirical FDR was defined as the proportion of permutations in which the number of significant results was greater than or equal to that observed in the experimental data.

### 2.8 Ontology analysis

Gene set enrichment analysis of the 95 risperidone-regulated genes was performed using the Enrichr web tool (RRID:SCR_001575, Kuleshov et al., 2016) and the Enrichr-KG platform (Evangelista et al., 2023). We assessed overrepresentation of gene annotations in the GeDiPNet 2023 library available in Enrichr, which integrates major public resources reporting human gene–disease associations and includes 2,388 disease terms spanning both somatic and psychiatric conditions. Statistical significance was determined using Fisher’s exact test and adjusted using the Benjamini–Hochberg method to correct for multiple hypothesis testing. Functional relationships among the 18 risperidone-regulated genes associated with schizophrenia were assessed using the Enrichr-KG tool (Evangelista et al., 2023). Analyses were conducted using four annotation sets: ChEA3 2022 (transcription factor regulation), MGI Mammalian Phenotype Level 42,021 (phenotypic annotations), GO Biological Processes 2021 (biological functions), and Reactome 2022 (signaling pathways).

## 3 Results

### 3.1 Spatial patterns of gene expression in the forebrain

**Figure 1:**
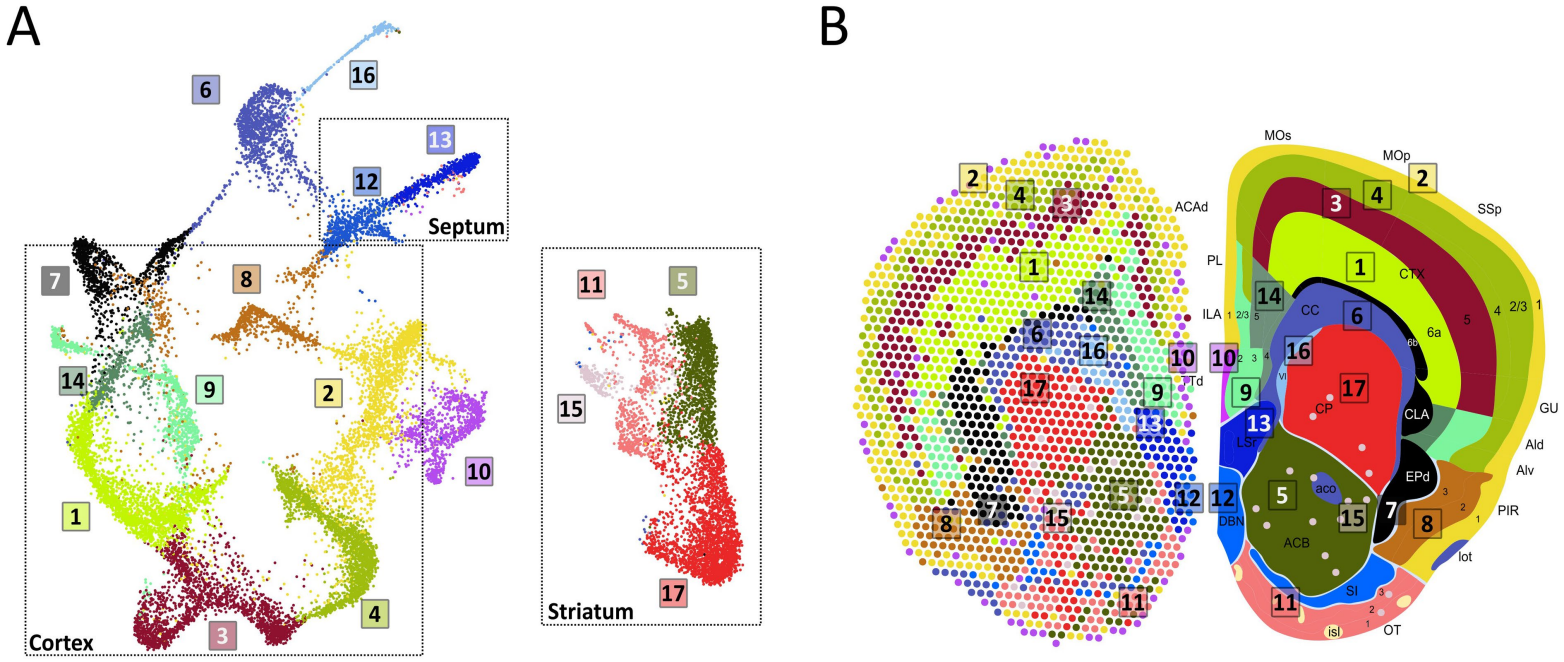
Spatial mapping of structural gene expression in the murine forebrain for region-specific drug effects analysis. **(A)** UMAP projection of unsupervised clustering of gene expression profiles corresponding to all individual spots. Colors represent spots constituting each of the 17 clusters. **(B)** Left: Spatial localization of spots in a representative brain section, with colors depicting cluster identities. Right: Coronal section of the mouse brain adapted from the Allen Reference Atlas – Mouse Brain. 1, 2, 3, 4, 5, 6a, 6b – layers of the cortex, ACAd- anterior cingulate area, dorsal part; ACB – nucleus accumbens; aco – anterior commissure; Ald – agranular insular cortex, dorsal part; Alv – agranular insular cortex, ventral part; CLA - claustrum; CP – caudatoputamen (dorsal striatum); CTX – cerebral cortex, DBN - diagonal band nuclei; EPd – endopiriform nucleus, dorsal part; fa – anterior forceps of the corpus callosum; GU – gustatory cortex; ILA – intralimbic cortex; isl – Islands of Calleja; lot – lateral olfactory tract; LSr – lateral septal nucleus, rostral part; MOp – primary motor cortex; MOs – supplementary motor area; OT – olfactory tubercle; PIR – piriform cortex; PL – prelimbic cortex; SI – substantia innominata; SSp – primary somatosensory cortex; TTd – Taenia tecta, dorsal part; VL – lateral ventricle.

Risperidone effects on gene expression were assessed in coronal forebrain sections (1.54–1.18 mm from bregma) from 10-week-old male mice that received an acute i.p. injection of 0.5 mg/kg risperidone or saline 2 h before brain collection. Sections were processed to construct 12 cDNA libraries, 6 from animals treated with risperidone and 6 from controls. The average cDNA fragment size was 440 bp. Reads were aligned to the GRCm38 reference genome. To analyze the data, we first performed unsupervised clustering of profiles from all individual spots across all sections (19,473 spots) using Seurat v4.4.0. A resolution parameter of 0.4 was selected, resulting in 17 clusters (#1 to #17), the minimum number needed to recapitulate the neuroanatomical structure of the forebrain sections in our dataset. Mean cluster sizes ranged from 19 (cluster #15, basal ganglia medium spiny neurons (MSN)/cholinergic cells) to 193 (cluster 17, caudatoputamen) spots across sections and showed no significant difference between groups. A UMAP projection of the clustering results is shown in Figure 1A. Clusters representing the nucleus accumbens (#5), caudatoputamen (#17), basal ganglia MSN/cholinergic cells (#15), and olfactory tubercle (#11) are close together, consistent with the common presence of MSN in these regions. Conversely, the corpus callosum (#6), characterized by dense fiber tracts and a high content of oligodendrocytes and glia, is located near the diagonal band (#12) and lateral septal nucleus (#13), which also contain many fibers and oligodendrocytes. Clusters representing cortical divisions (#1–4, #7–10, #14) are also grouped in the UMAP projection.

As shown in Figure 1B, the spatial arrangement of the clusters in a representative section closely matches the anatomical organization of the forebrain. Consequently, clusters are enriched with structure-specific markers described in the literature, supporting their anatomical identity as listed in Supplementary Table 1. Based on their anatomical location and gene expression profiles, clusters #2 and #10 correspond to the brain meninges and cortical layer 1, characterized by numerous non-neuronal markers (DeSisto et al., 2020; Remsik et al., 2021). Clusters #4, #3, and #1 represent inner cortical layers: cluster #4 aligns with layers 2/3, marked by genes such as *Lamp5, Calb1* and *Rasgrf2* (Maynard et al., 2021); cluster #3 corresponds to layers 4/5, characterized by *Lmo4 and S100a10* expression (Maynard et al., 2021); and cluster #1 matches layers 5/6, with markers like *Pde1a, Rprm, Dkk3* (Del-Aguila et al., 2019; Maynard et al., 2021). Clusters #9 and #14 include the medial prefrontal cortex, prelimbic and infralimbic areas, taenia tecta, insular cortex, and gustatory areas, expressing genes such as *Enc1, Fezf2* in cluster #9, and *Fezf2, Nxph3, Tle4 in #14* (Cahoy et al., 2008; Skene et al., 2018; Salem et al., 2024). Cluster #8 likely represents the ventral layers of the piriform cortex, characterized by inhibitory interneuron markers, including *Lmo3, Sst*, and *Cdhr1* [based on the interneuron marker list from PanglaoDB (Franzén et al., 2019)]. The claustrum, endopiriform nucleus, and cortical layer 6b are grouped within cluster #7, which expresses genes such as *Nxph4*, *Nr4a2*, and *Adra2a* (Del-Aguila et al., 2019). Cluster #6 comprises the corpus callosum, anterior commissure, and lateral olfactory tract, identified by oligodendrocyte markers like *Mog*, *Mbp*, *Opalin*, and *Mobp* (Horiuchi et al., 2017; Skene et al., 2018). Adjacent to the corpus callosum, cluster #16 corresponds to the subventricular zone, indicated by neuronal progenitor markers such as *Igfbp5* and *Sox2* (Mizrak et al., 2019). Additionally, the analysis distinguished between the dorsal (cluster #17) and ventral parts of the striatum (clusters #5 and #11). Cluster #17 matches the caudatoputamen, expressing markers like *Adora2a, Drd2*, and *Kcnip2* (Skene et al., 2018). Cluster #5 corresponds to the nucleus accumbens and expresses medium spiny neuron markers, including *Drd1*, *Drd2*, *Bcl11b*, and *Adora2a.* Cluster #15 likely represents basal ganglia MSN/cholinergic cells expressing MSN markers mentioned above (e.g., *Drd1* and *Drd2*) along with cholinergic cell markers – *Ache, Chat, Ecel1* (Skene et al., 2018). Cluster #11 is identified as the olfactory tubercle based on the expression of genes such as *Otof1*, *Pcp4*, and *Ppp1r2* [list compiled by Diamant et al. (2025) from the Allen Atlas and Lein et al. (2007)]. Medial to the striatum, cluster #12 corresponds to the diagonal band nucleus, marked by *Lhx6*, *Rab3b*, *Elfn1*, and *Gad2* (Diamant et al., 2025), while the adjacent cluster #13 represents the lateral septal nucleus, enriched in *Ecel1*, *Gfra1*, and *Cxcl14* (Diamant et al., 2025).

### 3.2 Drug-induced differential gene expression

**Figure 2:**
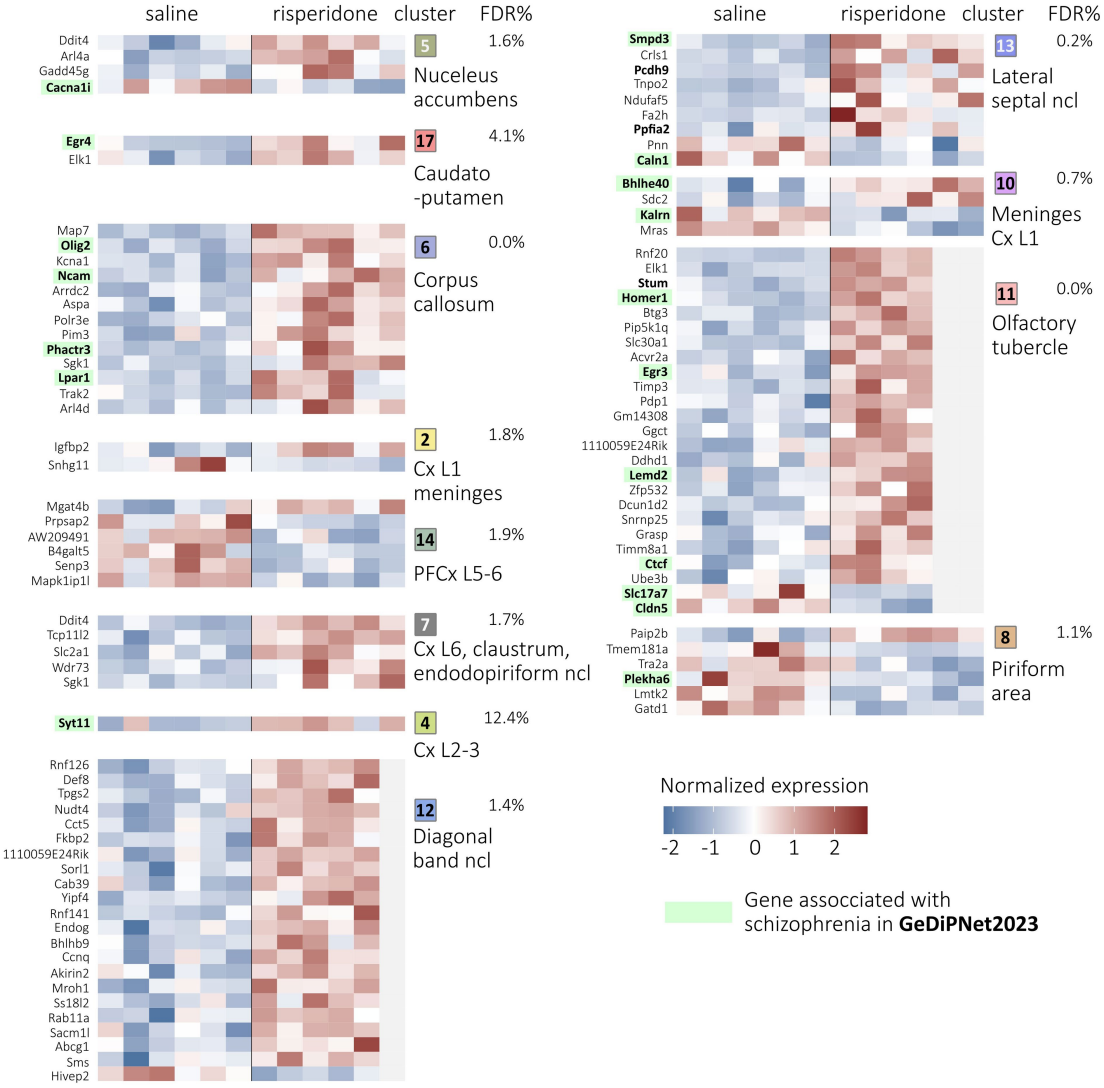
Risperidone-induced gene expression alterations in the murine forebrain. RNA-seq results with spatial localization (left) are presented as heatmaps showing 95 transcripts with transcriptome-wide significance (% FDR permutation-based per cluster). The heatmap illustrates transcript abundance 2 hours after risperidone injection, with color intensity proportional to the standardized value (z-score between −2 and 2) as indicated on the adjacent color scale. Each column corresponds to one animal for a total of 12 mice. The full list of 95 drug-affected transcripts is shown on the left. Subset of 18 genes enriched for the schizophrenia term in the GeDiPNet 2023 database (*p* = 1.71 × 10^−⁴^, q-value) is highlighted in green.

**Figure 3:**
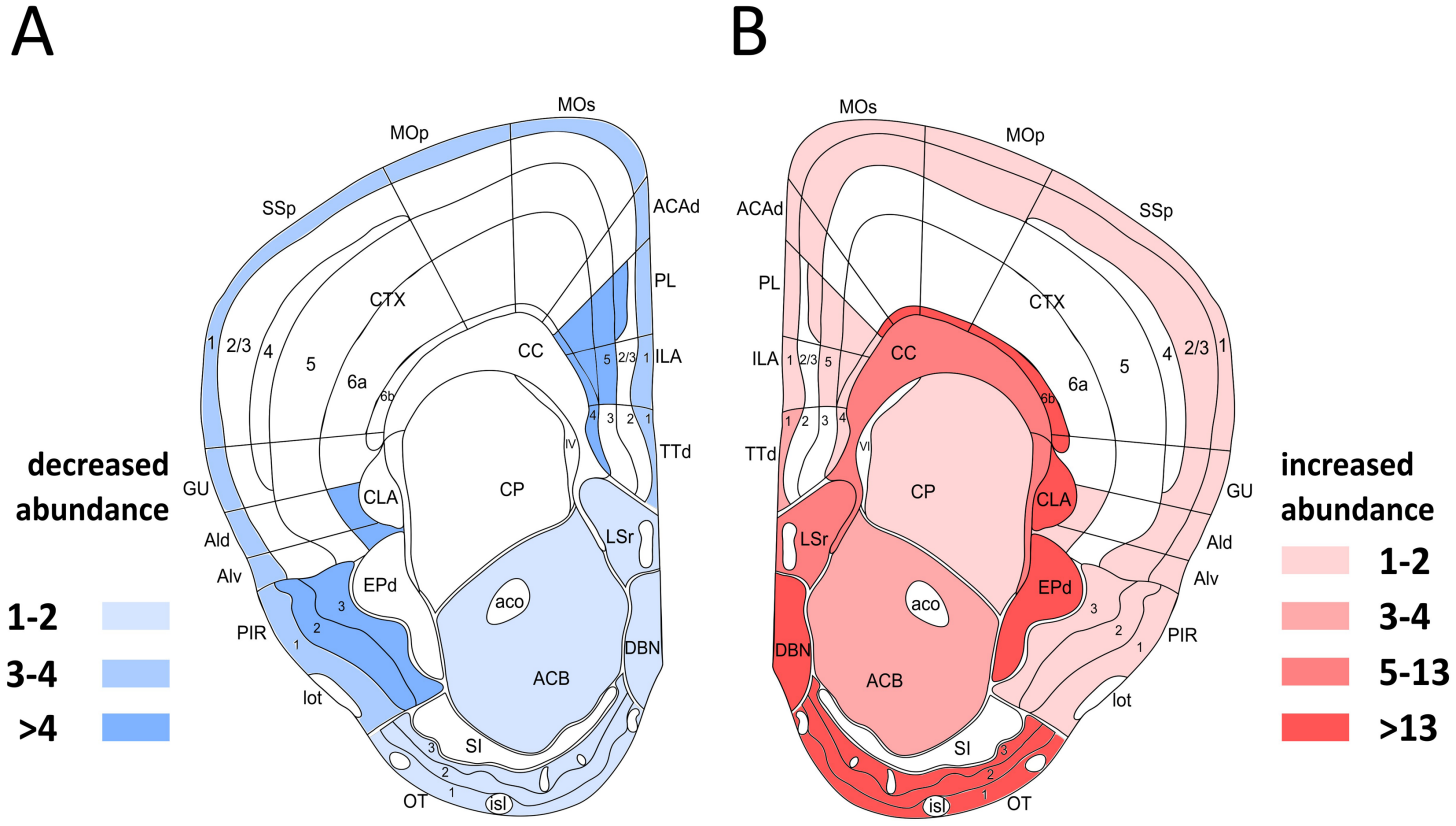
Regional distribution of 95 differentially expressed genes mapped onto a coronal section of the mouse brain, adapted from the Allen Reference Atlas – Mouse Brain. **(A)** The number of downregulated transcripts per brain region. **(B)** The number of upregulated transcripts per brain region. Color intensity reflects the number of transcripts with altered abundance within each cluster.

Differential gene expression caused by acute risperidone treatment was assessed within each cluster. Transcript abundance was normalized using quantile normalization across brain sections, and average expression levels were calculated for all spatial spots assigned to each cluster per section. Differential expression between treatment groups was evaluated using the Mann–Whitney U test. Genes were considered significant if they exhibited a |log₂(fold change)| ≥ ±0.8, a *p*-value < 0.01, and a minimum mean transcript abundance of 0.2 in at least one group. A total of 95 genes met these criteria, including 76 upregulated and 19 downregulated following risperidone treatment (Supplementary Table 2). Four genes—*Ddit4*, *Elk1*, *Sgk1*, and *1110059E24Rik (C9orf85)*—showed regulation in multiple regions, while the remaining 91 genes exhibited significant abundance changes in a single structure. Significant differential expression of specific transcripts was observed in 12 of 17 clusters (Figure 2). False discovery rates were calculated separately for each cluster to account for multiple testing and are listed next to the respective clusters in Figure 2. Most gene expression changes were seen in subcortical areas (Figure 3). The greatest number of changes was found in the olfactory tubercle (#11) with 25 transcripts regulated by risperidone, including activity-responsive genes such as *Homer1*, *Egr3*, and *Elk1*. Next was the diagonal band nucleus (#12) with 22 risperidone-regulated genes, including *Sorl1* and *Rab11a*. There were 9 differentially expressed transcripts in the adjacent lateral septal nucleus (#13), including *Smpd3* and *Fah2*. A total of 13 genes were upregulated in the brain commissures (#6), including *Ncam1* and *Kcna1*. Several oligodendrocyte markers, such as *Olig2* and *Lpar1*, were also elevated.

Taken together, the largest number of expression changes occurred in glia-enriched regions - including the commissures (#6), diagonal band (#12), lateral septal nucleus (#13), and olfactory tubercle (#11) - with most genes showing increased transcript levels (Figure 3). Conversely, cortical regions such as dorsal cortical layers 5 and 6 (#1), the piriform cortex (#8), and top cortical layers and meninges (#2, #10) primarily showed downregulated gene expression following risperidone exposure. Interestingly, the dorsal and ventral striatum exhibited relatively few significant changes, despite having the highest levels of dopamine receptor D2 expression, which is the main pharmacological target of risperidone. In the dorsal striatum (#17), only two activity-dependent transcription factors, *Egr4* and *Elk1*, were significantly induced, while in the nucleus accumbens (#5), changes in the expression of *Ddit4*, *Arl4a*, *Gadd45g*, and *Cacna1i* were noted.

### 3.3 Enrichment analysis

**Figure 4:**
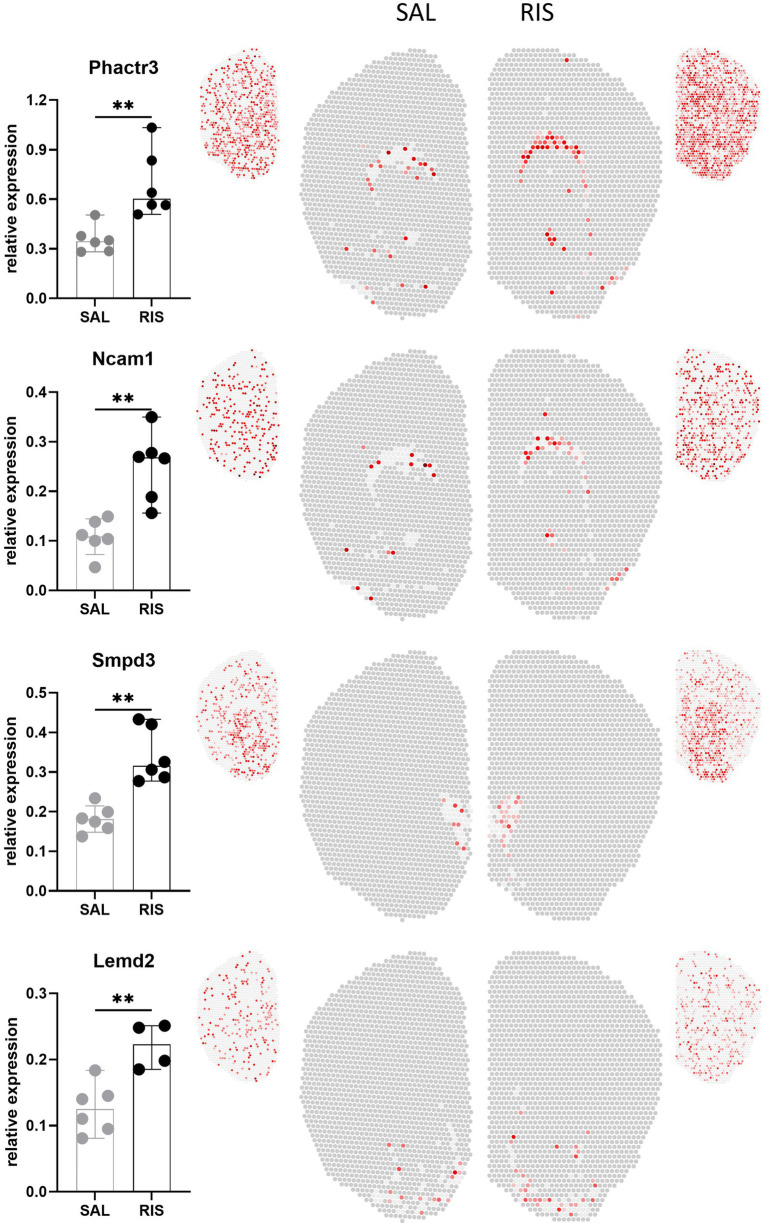
Spatial gene expression profiles of transcripts with increased abundance following acute risperidone treatment. The bar plots (left) and spatial gene expression profiles (right) illustrate the relative expression levels of genes upregulated by risperidone and associated with schizophrenia in GeDiPNet 2023 database. Bar plots show medians with 95% confidence intervals, based on Mann–Whitney U test results (**p < 0.01). Full expression profiles are displayed alongside the main images. Statistically significant changes were observed only in specific clusters, as outlined in the main panels.

**Figure 5:**
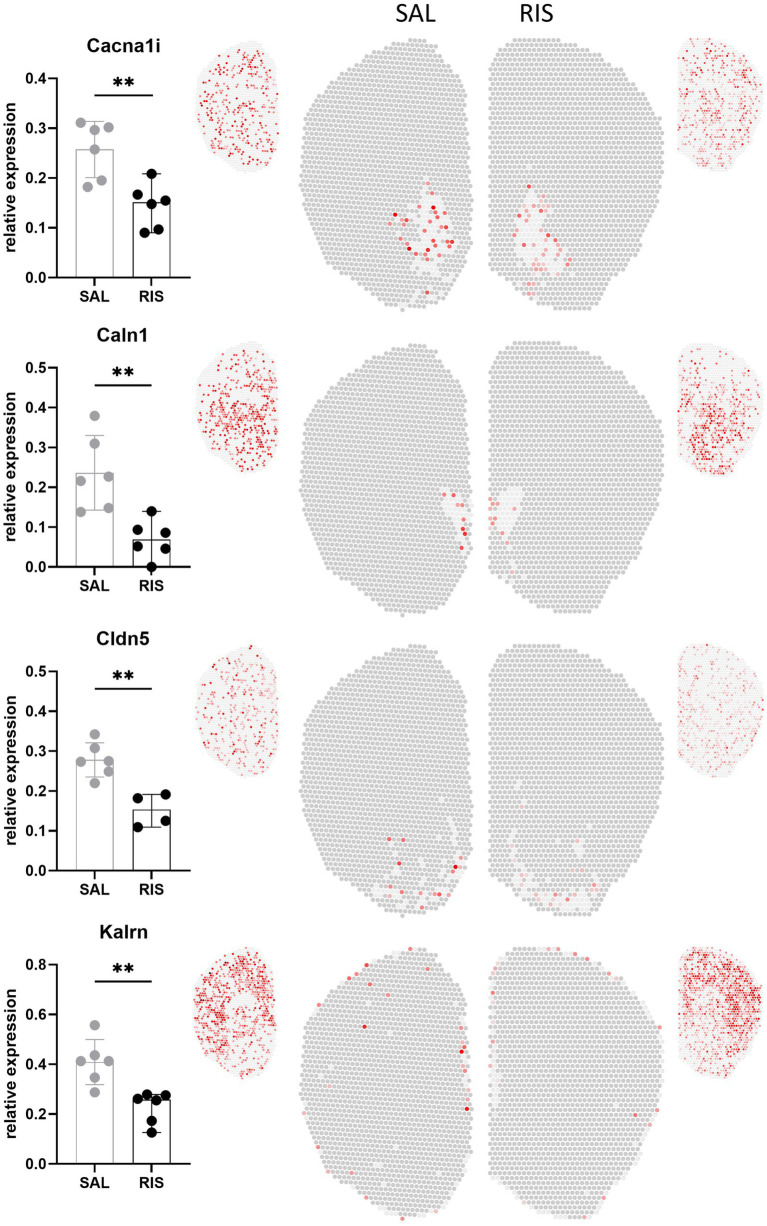
Spatial gene expression profiles of transcripts with decreased abundance following acute risperidone treatment. The bar plots (left) and spatial gene expression profiles (right) illustrate the relative expression levels of genes downregulated by risperidone and associated with schizophrenia in GeDiPNet 2023 database. Bar plots show medians with 95% confidence intervals, based on Mann–Whitney U test results (**p < 0.01). Full expression profiles are displayed alongside the main images. Statistically significant changes were observed only in specific clusters, as outlined in the main panels.

We have performed enrichment analysis on the 95 risperidone-regulated genes using Enrichr and annotations from the Gene Disease Pathway Networks database [“GeDiPNet 2023,” (Kundu et al., 2023)] that integrates data from all major clinical databases (including DisGeNET, ClinGen, ClinVar, HPO, OrphaNet, and PsyGeNET). ‘Schizophrenia’ emerged as the top-ranked term with the largest annotation overrepresentation—18 of the 95 risperidone-regulated transcripts associated with this condition (Fisher’s exact test, *p* = 1.71 × 10^−4^, q = 0.0568; Supplementary Table 3). These included *Egr3*, *Homer1*, *Cldn5*, *Lemd2*, *Slc17a7*, and *Ctcf* in the olfactory tubercle (#11); *Egr4* in the caudatoputamen (#17); *Phactr3*, *Olig2*, *Ncam1*, and *Lpar1* in the corpus callosum (#6); *Plekha6* in the piriform cortex (#8); *Bhlhe40* and *Kalrn* in the meninges/layer 1 of the cortex (#2); *Smpd3* and *Caln1* in the lateral septal nucleus (#13); *Cacna1i* in the nucleus accumbens (#5); and *Syt11* in cortical layers 2 and 3 (#4). Brain region-specific changes in the expression of risperidone-regulated genes enriched for schizophrenia in the GeDiPNet 2023 are presented in Figure 4 (increases in abundance) and Figure 5 (decreases in abundance). *Phactr3* (Ikeda et al., 2010; Li et al., 2017) and *Ncam1* (Trubetskoy et al., 2022; Dang et al., 2025) were upregulated in the areas corresponding to the commissures (#6, Figure 4), *Smpd3* (Trubetskoy et al., 2022) in the lateral septal nucleus (#13), and *Lemd2* (Trubetskoy et al., 2022) in the olfactory tubercle (#11). In turn, *Cacna1i* (Li et al., 2017; Lam et al., 2019) had reduced expression in the nucleus accumbens (#5, Figure 5), *Caln1* (Li et al., 2017; Lam et al., 2019) was decreased in the lateral septal nucleus (#13), and *Kalrn* (Ikeda et al., 2010; Li et al., 2017) in the top cortical layers and/or the meninges (#2).

**Figure 6:**
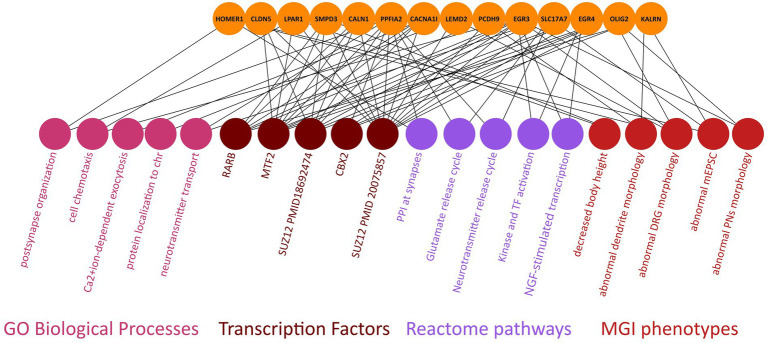
Overview of functional connections among risperidone-regulated genes linked to schizophrenia. The knowledge graph displays associations with enriched categories: transcription factor regulatory elements from ChEA3 2022 (maroon), morphological and functional phenotypes from MGI Mammalian Phenotype Level 4 2021 (red), biological processes from GO Biological Processes 2021 (pink), and cellular pathways from Reactome 2022 (purple). The graph was prepared based on results obtained with the Enrichr-KG tool. The complete list of terms is enclosed in Supplementary Table 3.

To investigate the functional roles of the 18 schizophrenia-associated genes acutely regulated by risperidone in naïve animals, we performed targeted enrichment analysis using Enrichr-KG (Evangelista et al., 2023). Results are shown in Figure 6, with the complete list provided in Supplementary Table 4. Transcription factor binding analysis revealed significant enrichment of Polycomb group proteins, including SUZ12 (13 promoters; *q* = 4.019 × 10^−4^), MTF2 (10 promoters; *q* = 3.704 × 10^−3^), and CBX2 (5 promoters; *q* = 1.426 × 10^−2^). Gene Ontology analysis indicated enrichment in regulation of calcium ion–dependent exocytosis (GO:0017158; *Cacna1i, Syt11*; *q* = 4.810 × 10^−2^) and response to calcium ion (GO:0051592; *Homer1, Syt11*; *q* = 8.528 × 10^−2^). Reactome pathway analysis linked *Egr3* and *Egr4* to NGF-stimulated transcription (R-HSA-9031628; *q* = 4.724 × 10^−2^), kinase and transcription factor activation (R-HSA-198725; *q* = 5.766 × 10^−2^), and NTRK1 signaling (R-HSA-187037; *q* = 8.283 × 10^−2^). MGI phenotype analysis further associated *Homer1, Slc17a7*, and *Kalrn* with abnormal miniature excitatory postsynaptic currents (MP:0004753; *q* = 7.857 × 10^−3^), and *Homer1, Slc17a7*, and *Egr3* with abnormal nervous system physiology (MP:0003633; *q* = 9.931 × 10^−3^). Together, these findings indicate enrichment for Polycomb-mediated transcriptional regulation and a possible convergence on calcium-dependent synaptic and NGF–NTRK1 signaling pathways implicated in neuronal plasticity.

## 4 Discussion

Two key insights emerge from the spatial analysis of drug-regulated gene expression patterns. First, transcriptional responses to acute risperidone treatment in male mice are specific to brain regions, with distinct sets of genes regulated in adjacent structures. Second, there is a notable overrepresentation of genes related to schizophrenia genetics among the differentially regulated transcripts, showing that antipsychotic treatment in healthy naïve animals affects molecular pathways involved in the disease’s pathophysiology.

We observed strong risperidone-induced changes in transcript levels in subcortical regions of the forebrain, which largely align with prior findings on gene expression changes following antipsychotic treatment in animal models (Chen and Chen, 2005; Fehér et al., 2005; Korostynski et al., 2013; De Bartolomeis et al., 2017; Zygmunt et al., 2019) and post-mortem samples from schizophrenia patients (Merikangas et al., 2022; Amjad and Sokouti, 2025) Several genes that are differentially expressed, including *Bhlhe40*, *Egr3*, *Egr4*, *Elk1*, *Sgk1*, *Ddit4*, and *Homer1a*, are activity-dependent transcripts previously reported to be acutely regulated by antipsychotics in the striatum, cortex, and corpus callosum. Most of these changes occurred in the septal area, as well as in the olfactory tubercle and piriform cortex, rather than in the nucleus accumbens or prefrontal cortex. The potential involvement of the medial septal nuclei, and in particular the diagonal band nucleus, could be of particular interest, as these have a major role in the control of the theta rhythms in the hippocampus and formation of episodic memories (Buzsáki, 2002; Goutagny et al., 2008; Lega et al., 2012). Thus, speculatively, these effects could contribute to the long-term effects of antipsychotics on cognitive functions.

Some well-studied activity-regulated transcripts in the context of antipsychotics, such as *Fos*, *Fosb*, or *Egr1*, are absent from our results. The overall difference in spatial patterns likely stems from the historical focus on preselected brain regions directly involved in dopaminergic pathology in schizophrenia, as well as findings from *in situ* hybridization experiments assessing *Fos* expression. Notably, early studies reported antipsychotic-induced changes in gene expression in the olfactory tubercle, septum, and ventral cortical regions (Robertson and Fibiger, 1992; Cools et al., 1995). However, these findings were not followed up with extensive research. The pattern of risperidone-induced gene expression overlaps only slightly with the distribution of dopamine D2 and serotonin 5-HT2A receptors. In the forebrain, D2 receptors are mainly found in a subset of medium spiny neurons within the striatum, nucleus accumbens, and olfactory tubercle, as well as in the upper layers of the cortex (Meador-Woodruff et al., 1989; Gangarossa et al., 2013; Cieslak et al., 2024). Conversely, 5-HT2A receptors are widely expressed in the cortex—including the claustrum and piriform cortex—but are mostly absent from the basal ganglia and septum (Burnet et al., 1995). Cellular co-expression of D2 and 5-HT2A receptors is rare, but functional interactions between these receptors in the striatum may help reduce extrapyramidal side effects of D2 antagonists (Bubser et al., 2001). In our study, apart from the olfactory tubercle, the regions displaying the most extensive risperidone-induced transcriptional changes also exhibited low levels of D2 and 5-HT2A receptor expression—such as the septum, corpus callosum, and commissures—indicating that these effects are likely indirect. Similar results were recently observed with chronic olanzapine treatment (Abrantes et al., 2022), where differentially expressed genes were not enriched for dopaminergic pathways despite known interactions with D2-expressing neurons. It should be noted, though, that risperidone also acts as an antagonist of other monoamine receptors, including dopamine receptors D3 and D4, adrenergic α2 receptors, and histamine H1 receptors, and it is possible that they could be responsible for the drug effects in brain areas with relatively low D2 and 5-HT2A expression (Schotte et al., 1996). These observations show a potential role for glial cells in early transcriptional responses to risperidone. However, whether these effects persist or change with chronic treatment remains to be determined.

We observed a statistically significant overlap between transcripts acutely regulated by risperidone in naïve male mice and genes associated with schizophrenia in a non-targeted analysis using the GeDiPNet 2023 database. Schizophrenia emerged as the top-ranked annotation, with the largest gene count among all somatic, neurological, and psychiatric conditions. This finding is consistent with recent reports linking antipsychotic-regulated genes to the etiology of schizophrenia (Abrantes et al., 2022; Chestnykh et al., 2025). Several of the identified genes have previously been implicated in schizophrenia in human genome-wide association studies reported in the NHGRI-EBI GWAS Catalog (Cerezo et al., 2025). These include *Smpd3, Ppfia2, Caln1,* and *Pcdh9* (lateral septal nucleus, cluster #13); *Cacna1i* (nucleus accumbens, cluster #5); *Phactr3* and *Ncam1* (corpus callosum, cluster #6); *Lemd2* and *Stum* (olfactory tubercle, cluster #11); and *Kalrn* (meninges and superficial cortical layers, cluster #10), as associated with schizophrenia.

We demonstrate that the schizophrenia-related transcripts induced by risperidone in naïve mice are not uniformly distributed across the forebrain. Many disease-related genes exhibit differential expression regulation in the olfactory tubercle, lateral septal nucleus, and brain commissures, which together account for 12 of the 18 schizophrenia-linked genes. Notably, risperidone increased the expression of *Olig2* in the corpus callosum and anterior commissure (#6), which was previously found to have reduced expression in the brains of schizophrenic and bipolar patients (Tkachev et al., 2003). *Olig2,* as a key transcription factor, may affect the expression of other myelin-related genes, as it influences precursor and mature oligodendrocytes and is both necessary and sufficient for oligodendrocyte generation and myelination (Georgieva et al., 2006). The downregulation of *Cacna1i* in the nucleus accumbens (#5) after risperidone administration is also of particular interest, given this region’s role in motivation, emotions, and limbic–motor integration (Salamone and Correa, 2012). *Cacna1i* encodes the Cav3.3 T-type calcium channel, which contributes to neuronal rhythmic firing. Elevated *CACNA1I* expression has been reported in the hippocampus of schizophrenia patients (Xie et al., 2018), suggesting that risperidone may modulate disease-associated transcriptional changes. However, further studies, particularly in human tissue, are needed to confirm whether similar effects occur in the nucleus accumbens of patients treated with antipsychotics. Another interesting observation is the increased expression of *Smpd3* in the lateral septal nucleus (#13) following risperidone treatment. *Smpd3* encodes neutral sphingomyelinase-2 (NSM), an enzyme involved in sphingolipid metabolism and synaptic development. Abnormal sphingolipid metabolism has been implicated in schizophrenia and comorbid metabolic syndrome (Castillo et al., 2016; Chestnykh et al., 2025), though the specific role of *SMPD3* in cognitive decline and neurodegeneration remains unclear (Stoffel et al., 2018; Zhu et al., 2024). Finally, the enrichment revealed that schizophrenia-associated genes regulated by risperidone in healthy mice had increased frequency of binding sites for Polycomb group proteins, including SUZ12, MTF2, and CBX2. Disrupted Polycomb complex activity is associated with the developmental disorders (e.g., Cyrus et al., 2019), which makes it attractive to speculate that it may play a yet unrecognized role in coordinating antipsychotic response in the brain. However, we note that the observed changes in expression do not necessarily colocalize in the same cell types, and thus, potential common regulatory mechanisms need to be interpreted with caution.

Our study has multiple limitations. First, the model uses naïve animals and a single acute dose of 0.5 mg/kg risperidone. Because this study was performed in healthy wild-type mice, the observed transcriptional responses should be interpreted as a baseline characterization of acute risperidone effects rather than as a direct model of its therapeutic mechanisms in schizophrenia. Nevertheless, establishing these baseline effects is a necessary step before extending this spatial transcriptomic approach to more complex disease-relevant models. We have previously demonstrated that the same model and drug dose are effective in analyzing the mechanisms of various psychotropic drugs (Korostynski et al., 2013). Previous reports showed that it was in the range of doses that did not impair behavioral responses to stimuli or cause appreciable sedation (Le Pen and Moreau, 2002; Baba et al., 2019). Moreover, it is debatable how well schizophrenia or its symptoms can be modeled in rodents (Powell and Miyakawa, 2006). New mouse models carrying mutations equivalent to rare human mutations associated with a very high risk of schizophrenia may provide a better approximation of the underlying pathology (Meechan et al., 2015; Rutkowski et al., 2021). However, analyzing differential gene expression with spatial resolution is a novel approach—the methodology is still evolving, and previous data, especially on the effects of acute antipsychotic treatment in naïve animals, have been largely unavailable. The present study aimed to fill this gap by offering one of the first brain-wide spatial transcriptomic datasets capturing gene expression responses to drug treatment. A second major limitation of our study stems from the technical constraints of the method. Limited sequencing depth per tissue spot biases the dataset toward more abundant transcripts. The absence of *Fos* and several other activity-regulated transcripts may reflect sequencing limitations or the exclusion of low-abundance transcripts while handling a multidimensional, zero-inflated dataset. The spatial resolution is not at the single-cell level, resulting in a variable number of cells per spot, which further complicates interpretation. This is especially relevant for antipsychotics, as their primary target, the D2 receptors, are expressed in neurons within highly heterogeneous structures. For example, in the striatum, D2- and D1-expressing medium spiny neurons are distinct but notoriously difficult to separate, and often have opposing functions, such as mediating the effects of antipsychotics and psychostimulants (Bertran-Gonzalez et al., 2008). Therefore, a single spot in our dataset may contain a mixture of functionally different neurons, glia, and other cell types. This challenge may be addressed through methodological improvements and increased spatial resolution, although this will increase analytical complexity. While the observed expression patterns are unlikely to reflect the full range of transcriptional changes, they nevertheless enable region-specific assessment of risperidone effects and provide insight into the underlying mechanisms. Third, this study assessed only the acute effects of risperidone and therefore may not reflect chronic adaptations in patients. We focused on acute treatment to enable comparison with previous reports and evaluate the accuracy and strengths of the spatial approach. Because endpoints in chronic studies can be confounded by recent dosing or withdrawal effects, our aim at this stage was to establish and validate the methodology for future studies of antipsychotic-related neuronal mechanisms. Finally, we examined only 10-week-old male mice, as this time point corresponds to the peak risk period for schizophrenia onset in men (McGrath et al., 2004; Giordano et al., 2021). Nevertheless, extending the study to include females is essential because sex differences in schizophrenia incidence, clinical course, and antipsychotic response may reflect sex-specific transcriptional responses to risperidone. Such studies will need to account for estrous cycle–dependent fluctuations in gonadal hormones, which can affect dopamine signaling, gene expression, schizophrenia risk, and drug response. At the current stage, the absence of females limits the ability to generalize these findings and precludes assessment of sex-specific effects.

In summary, the spatial analysis of risperidone-regulated gene expression in the mouse forebrain offers significant new insights into the mechanisms of antipsychotic drug action. It demonstrates a notable overrepresentation of schizophrenia-associated genes among transcripts acutely regulated by risperidone in naïve male mice. Further research using models with stronger construct validity, chronic treatment paradigms, and higher-resolution single-cell spatial transcriptomics is needed to validate these mechanisms and clarify their relevance to long-term antipsychotic efficacy.

## Data Availability

The RNAseq dataset generated for this study can be found in the Sequence Read Archive, https://www.ncbi.nlm.nih.gov/, under accession number PRJNA1143882.

## Footnotes

1
https://github.com/macs3-project/MACS

2
https://github.com/ippas/ifpan-janrod-spatial
