# Supplementary material for: Risperidone regulates the expression of schizophrenia-related genes in the forebrain of adult male mice
Source: Front Mol Neurosci. 2026 May 29;19:1844705. doi: 10.3389/fnmol.2026.1844705 (PMC13260059; doi:10.3389/fnmol.2026.1844705)
Supplement: Supplementary file 1 [file Table_1.DOCX]

Supplementary Table 1

Identification of structural and cell-type markers based on literature and database sources.

| Cluster | Structure | Markers | Source |
| --- | --- | --- | --- |
| 1 | Cx L5-6A | Layers 5-6 [RPRM, IGFBP4, DKK3, NEFH, TBR1, PDE1A] | (Del-Aguila et al., 2019; Maynard et al., 2021) |
| 2 | Cortex layer 1/Meninges | Astrocytes [APOE, CST3 CPE, MT3] | (DeSisto et al., 2020; Diamant et al., 2025)  Astrocyte Gene Set |
| 3 | Cortex layers 4-5 | Layers 4-5  [MEF2C, SNAP25, S100A10, LMO4] | (Maynard et al., 2021) |
| 4 | Cortex layers 2-3 | Layers 2-3  [CALB1, RASGRF2, LAMP5, MEF2C] | (Del-Aguila et al., 2019; Maynard et al., 2021) |
| 5 | Nucleus accumbens | [DRD1, DRD2, PENK, CRYM, BCL11B, ADORA2A] | (Puighermanal et al., 2020) |
| 6 | Corpus callosum/anterior commissure | Oligodendrocytes [MOG, TSPAN2, MBP, PLEKHH1, ERMN, PLEKHB1, EVI2A, APOD, FA2H, MAL, PDLIM2, OPALIN, MOBP, ANLN, PLLP, PLP1] | (Skene et al., 2018;  Horiuchi et al., 2017) |
| 7 | Cortex layer 6b/ Claustrum/ Endopiriform nucleus | [CCN2, ADRA2A, NXPH4, NR4A2, CADPS2, GNB4, GNG2] | (Del-Aguila et al., 2019) |
| 8 | Piriform area | Interneurons [LMO3, CDHR1, NPTXR, RAB3B, SST] | (Franzén et al., 2019) PanglaoDB Augmented 2021 |
| 9 | prelimbic and infralimbic area, medial PFC layers 2-3 | Pyramidal cells [CCK, FEZF2, ENC1, DOC2A, C1QL3, MEF2C] | (Skene et al., 2018) |
|  |  | Neurons [CCK, FEZF2, ENC1, DOC2A, C1QL3, MEF2C, GRP] | (Cahoy et al., 2008) |
| 10 | Meninges/Cortex layer 1 | [SLC38A2, MGP, FXYD5] | (DeSisto et al., 2020; Remsik et al., 2021) |
| 11 | Olfactory tubercle | [PCP4, OTOF, PPP1R2, GNG7, OSBPL8] | (Diamant et al., 2025) Olfactory tubercle set |
| 12 | Diagonal band nucleus | [LHX6, RAB3B, ELFN1, ACHE, GAD2] | (Diamant et al., 2025) Diagonal band nucleus set |
| 13 | Lateral septal nucleus | [ECEL1, GFRA1, CXCL14] | (Diamant et al., 2025) Lateral septal nucleus set |
| 14 | medial PFC layers 5-6 | [PDE1A, NXPH3, TLE4, FXYD6]  [RPRM, FEZF2] | (Salem et al., 2024)  (Maynard et al., 2021)) |
| 15 | basal ganglia MSN/cholinergic cells | medium spiny neurons [DRD2, PENK, ADORA2A, DRD1]  striatal interneurons [CHAT, ACHE, ECEL1] | (Skene et al., 2018) |
| 16 | SVZ | Neuronal progenitor cells [SOX2, IGFBP5, CPE] | (Mizrak et al., 2019)  ARCHS4 Tissues Gene Set |
| 17 | Caudatoputamen | Medium Spiny Neurons  [PENK, ADORA2A, DRD2, RELN, KCNIP2, DMKN, RXRG, CD4, CALB1, DGKB, SYNPR, KCNK2] | (Skene et al., 2018) |
