# Supplementary material for: Risperidone regulates the expression of schizophrenia-related genes in the forebrain of adult male mice
Source: Front Mol Neurosci. 2026 May 29;19:1844705. doi: 10.3389/fnmol.2026.1844705 (PMC13260059; doi:10.3389/fnmol.2026.1844705)
Supplement: Supplementary file 2 [file Table_2.DOCX]

Supplementary Table 2

Differential expression analysis of 95 genes using our customised spaceranger-MACS3-Nanopore-spaceranger processing pipeline (see Methods for details). Fold change (log₂ ratio) and significance (Mann–Whitney U test) were calculated for each gene–cluster pair.

| Gene symbol | Cluster_nr | Structure | p value | log2FC |
| --- | --- | --- | --- | --- |
| Igfbp2 | cluster_2 | Cortex layer 1/Meninges | 0.0087 | 0.8945 |
| Snhg11 | cluster_2 | Cortex layer 1/Meninges | 0.0043 | -1.0492 |
| Syt11 | cluster_4 | Cortex layers 2-3 | 0.0043 | 1.0057 |
| Ddit4 | cluster_5 | Nucleus accumbens | 0.0043 | 0.9427 |
| Arl4a | cluster_5 | Nucleus accumbens | 0.0087 | 1.3141 |
| Gadd45g | cluster_5 | Nucleus accumbens | 0.0043 | 1.2566 |
| Cacna1i | cluster_5 | Nucleus accumbens | 0.0087 | -0.8369 |
| Trak2 | cluster_6 | Corpus callosum/anterior commissure | 0.0087 | 0.9613 |
| Map7 | cluster_6 | Corpus callosum/anterior commissure | 0.0022 | 0.9854 |
| Sgk1 | cluster_6 | Corpus callosum/anterior commissure | 0.0087 | 1.1385 |
| Aspa | cluster_6 | Corpus callosum/anterior commissure | 0.0022 | 0.8439 |
| Arl4d | cluster_6 | Corpus callosum/anterior commissure | 0.0087 | 1.7875 |
| Pim3 | cluster_6 | Corpus callosum/anterior commissure | 0.0087 | 1.0098 |
| Olig2 | cluster_6 | Corpus callosum/anterior commissure | 0.0022 | 1.2844 |
| Phactr3 | cluster_6 | Corpus callosum/anterior commissure | 0.0022 | 0.9544 |
| Lpar1 | cluster_6 | Corpus callosum/anterior commissure | 0.0087 | 1.0289 |
| Kcna1 | cluster_6 | Corpus callosum/anterior commissure | 0.0022 | 1.189 |
| Polr3e | cluster_6 | Corpus callosum/anterior commissure | 0.0043 | 1.4815 |
| Arrdc2 | cluster_6 | Corpus callosum/anterior commissure | 0.0022 | 1.9398 |
| Ncam1 | cluster_6 | Corpus callosum/anterior commissure | 0.0022 | 1.2131 |
| Sgk1 | cluster_7 | Cx layer 6b/ Claustrum/ Endopiriform nucleus | 0.0043 | 0.8875 |
| Ddit4 | cluster_7 | Cx layer 6b/ Claustrum/ Endopiriform nucleus | 0.0022 | 1.1824 |
| Tcp11l2 | cluster_7 | Cx layer 6b/ Claustrum/ Endopiriform nucleus | 0.0022 | 1.3059 |
| Slc2a1 | cluster_7 | Cx layer 6b/ Claustrum/ Endopiriform nucleus | 0.0043 | 0.8567 |
| Wdr73 | cluster_7 | Cx layer 6b/ Claustrum/ Endopiriform nucleus | 0.0087 | 0.9455 |
| Plekha6 | cluster_8 | Piriform area | 0.0043 | -0.8565 |
| Tmem181a | cluster_8 | Piriform area | 0.0022 | -1.175 |
| Lmtk2 | cluster_8 | Piriform area | 0.0087 | -1.486 |
| Tra2a | cluster_8 | Piriform area | 0.0087 | -0.8666 |
| Paip2b | cluster_8 | Piriform area | 0.0043 | 0.8692 |
| Gatd1 | cluster_8 | Piriform area | 0.0022 | -0.8724 |
| Sdc2 | cluster_10 | Meninges/Cortex layer 1 | 0.0087 | 0.8344 |
| Kalrn | cluster_10 | Meninges/Cortex layer 1 | 0.0022 | -0.8395 |
| Bhlhe40 | cluster_10 | Meninges/Cortex layer 1 | 0.0022 | 0.9891 |
| Mras | cluster_10 | Meninges/Cortex layer 1 | 0.0022 | -0.916 |
| Stum | cluster_11 | Olfactory tubercle | 0.0095 | 1.3731 |
| Slc30a1 | cluster_11 | Olfactory tubercle | 0.0095 | 0.9148 |
| Timp3 | cluster_11 | Olfactory tubercle | 0.0095 | 0.9267 |
| Snrnp25 | cluster_11 | Olfactory tubercle | 0.0095 | 0.8407 |
| Homer1 | cluster_11 | Olfactory tubercle | 0.0095 | 1.5232 |
| Ddhd1 | cluster_11 | Olfactory tubercle | 0.0095 | 0.8776 |
| Egr3 | cluster_11 | Olfactory tubercle | 0.0095 | 0.9738 |
| Grasp | cluster_11 | Olfactory tubercle | 0.0095 | 0.94 |
| Cldn5 | cluster_11 | Olfactory tubercle | 0.0095 | -0.8707 |
| Btg3 | cluster_11 | Olfactory tubercle | 0.0095 | 0.8459 |
| Lemd2 | cluster_11 | Olfactory tubercle | 0.0095 | 0.81 |
| Zfp532 | cluster_11 | Olfactory tubercle | 0.0095 | 1.144 |
| 1110059E24Rik | cluster_11 | Olfactory tubercle | 0.0095 | 1.031 |
| Acvr2a | cluster_11 | Olfactory tubercle | 0.0095 | 0.8587 |
| Gm14308 | cluster_11 | Olfactory tubercle | 0.0095 | 1.2875 |
| Pip5k1a | cluster_11 | Olfactory tubercle | 0.0095 | 0.8747 |
| Pdp1 | cluster_11 | Olfactory tubercle | 0.0095 | 0.8502 |
| Rnf20 | cluster_11 | Olfactory tubercle | 0.0095 | 0.8584 |
| Ube3b | cluster_11 | Olfactory tubercle | 0.0095 | 0.8643 |
| Ggct | cluster_11 | Olfactory tubercle | 0.0095 | 1.0947 |
| Slc17a7 | cluster_11 | Olfactory tubercle | 0.0095 | -1.3978 |
| Dcun1d2 | cluster_11 | Olfactory tubercle | 0.0095 | 0.8518 |
| Ctcf | cluster_11 | Olfactory tubercle | 0.0095 | 0.9814 |
| Elk1 | cluster_11 | Olfactory tubercle | 0.0095 | 1.2358 |
| Timm8a1 | cluster_11 | Olfactory tubercle | 0.0095 | 0.8603 |
| Cab39 | cluster_12 | Diagonal band nucleus | 0.0087 | 0.962 |
| Hivep2 | cluster_12 | Diagonal band nucleus | 0.0043 | -1.1231 |
| Rnf126 | cluster_12 | Diagonal band nucleus | 0.0043 | 0.8208 |
| Nudt4 | cluster_12 | Diagonal band nucleus | 0.0043 | 0.8664 |
| Ccnq | cluster_12 | Diagonal band nucleus | 0.0043 | 1.3251 |
| Cct5 | cluster_12 | Diagonal band nucleus | 0.0043 | 0.9483 |
| Mroh1 | cluster_12 | Diagonal band nucleus | 0.0043 | 1.2765 |
| Abcg1 | cluster_12 | Diagonal band nucleus | 0.0043 | 1.4173 |
| Yipf4 | cluster_12 | Diagonal band nucleus | 0.0043 | 0.9784 |
| Tpgs2 | cluster_12 | Diagonal band nucleus | 0.0043 | 0.9755 |
| Fkbp2 | cluster_12 | Diagonal band nucleus | 0.0043 | 0.8859 |
| 1110059E24Rik | cluster_12 | Diagonal band nucleus | 0.0043 | 1.4168 |
| Endog | cluster_12 | Diagonal band nucleus | 0.0043 | 0.9954 |
| Akirin2 | cluster_12 | Diagonal band nucleus | 0.0087 | 0.9107 |
| Rnf141 | cluster_12 | Diagonal band nucleus | 0.0043 | 1.3166 |
| Def8 | cluster_12 | Diagonal band nucleus | 0.008 | 2.1681 |
| Sorl1 | cluster_12 | Diagonal band nucleus | 0.0043 | 0.914 |
| Rab11a | cluster_12 | Diagonal band nucleus | 0.0087 | 0.8208 |
| Ss18l2 | cluster_12 | Diagonal band nucleus | 0.0087 | 0.8833 |
| Sacm1l | cluster_12 | Diagonal band nucleus | 0.0087 | 0.8379 |
| Bhlhb9 | cluster_12 | Diagonal band nucleus | 0.0087 | 0.964 |
| Sms | cluster_12 | Diagonal band nucleus | 0.0043 | 0.8698 |
| Ppfia2 | cluster_13 | Lateral septal nucleus | 0.0087 | 0.8457 |
| Pnn | cluster_13 | Lateral septal nucleus | 0.0087 | -0.8739 |
| Pcdh9 | cluster_13 | Lateral septal nucleus | 0.0022 | 0.9407 |
| Crls1 | cluster_13 | Lateral septal nucleus | 0.0043 | 1.3411 |
| Ndufaf5 | cluster_13 | Lateral septal nucleus | 0.0043 | 1.1133 |
| Caln1 | cluster_13 | Lateral septal nucleus | 0.0043 | -1.7662 |
| Tnpo2 | cluster_13 | Lateral septal nucleus | 0.0087 | 0.9455 |
| Smpd3 | cluster_13 | Lateral septal nucleus | 0.0022 | 0.9142 |
| Fa2h | cluster_13 | Lateral septal nucleus | 0.0043 | 0.8934 |
| Mgat4b | cluster_14 | Medial PFC layers 5-6 | 0.0087 | 0.869 |
| Prpsap2 | cluster_14 | Medial PFC layers 5-6 | 0.0087 | -1.036 |
| Senp3 | cluster_14 | Medial PFC layers 5-6 | 0.0043 | -1.4471 |
| AW209491 | cluster_14 | Medial PFC layers 5-6 | 0.0087 | -0.8497 |
| Mapk1ip1l | cluster_14 | Medial PFC layers 5-6 | 0.0087 | -1.0624 |
| B4galt5 | cluster_14 | Medial PFC layers 5-6 | 0.0087 | -0.8472 |
| Egr4 | cluster_17 | Caudatoputamen | 0.0043 | 1.4312 |
| Elk1 | cluster_17 | Caudatoputamen | 0.0043 | 0.9119 |
