# Supplementary material for: Risperidone regulates the expression of schizophrenia-related genes in the forebrain of adult male mice
Source: Front Mol Neurosci. 2026 May 29;19:1844705. doi: 10.3389/fnmol.2026.1844705 (PMC13260059; doi:10.3389/fnmol.2026.1844705)
Supplement: Supplementary file 3 [file Table_3.DOCX]

Supplementary Table 3

Enrichment analysis of risperidone-regulated genes for gene–disease associations in the GeDiPNet 2023 database, created with the Enrichr Appyter. The q-values are adjusted p-values calculated with the Benjamini–Hochberg method to correct for multiple hypothesis testing.

| Term | p-value | q-value | overlap genes |
| --- | --- | --- | --- |
| Schizophrenia | 0.000171 | 0.056766 | EGR3, HOMER1, EGR4, LPAR1, PLEKHA6, OLIG2, CTCF, KALRN, LEMD2, SMPD3, CLDN5, CACNA1I, SYT11, BHLHE40, PHACTR3, NCAM1, SLC17A7, CALN1 |
| Atrophy of the Spinal Cord | 0.000462 | 0.076398 | FA2H, CCT5 |
| Hypocholesterolemia | 0.001194 | 0.131751 | UBE3B, CCT5 |
| Clonic Seizures | 0.002351 | 0.141523 | KCNA1, SLC2A1, SLC30A1, SLC17A7 |
| Spastic Paraplegia | 0.002501 | 0.141523 | FA2H, SLC2A1, DDHD1, CCT5 |
| Rubral Tremor | 0.002565 | 0.141523 | EGR3, SLC2A1 |
| Hypotonic Seizures | 0.004254 | 0.197108 | KCNA1, SLC2A1, SLC30A1, SLC17A7 |
| Dyskinesia | 0.005757 | 0.197108 | HOMER1, SLC2A1 |
| Focal Seizures | 0.006238 | 0.197108 | FA2H, KCNA1 |
