# Supplementary material for: Risperidone regulates the expression of schizophrenia-related genes in the forebrain of adult male mice
Source: Front Mol Neurosci. 2026 May 29;19:1844705. doi: 10.3389/fnmol.2026.1844705 (PMC13260059; doi:10.3389/fnmol.2026.1844705)
Supplement: Supplementary file 4 [file Table_4.DOCX]

Supplementary Table 4

List of terms connected with risperidone-regulated, schizophrenia-associated 21 genes obtained by meta-analysis of these genes in Enrichr-KG tool using the following databases: ChEA3 2022, MGI Mammalian Phenotype Level 4 2021, GO Biological Processes 2021, and Reactome 2022.

| database | full term | abbreviated term in Figure 6. |
| --- | --- | --- |
| GO Biological Processes 2021 | response to calcium ion (GO:0051592) | response to Ca2+ ion |
| GO Biological Processes 2021 | cell chemotaxis (GO:0060326) | cell chemotaxis |
| GO Biological Processes 2021 | regulation of calcium ion-dependent exocytosis (GO:0017158) | Ca2+ ion-dependent exocytosis |
| GO Biological Processes 2021 | protein localization to chromosome (GO:0034502) | protein localization to chr |
| GO Biological Processes 2021 | phospholipase C-activating  G protein-coupled receptor signalling pathway (GO:0007200) | PLC-activating GPCR signaling |
| ChEA3 2022 | MTF2 20144788 ChIP-Seq MESCs Mouse | MTF2 |
| ChEA3 2022 | SIN3B 21632747 ChIP-Seq MESCs Mouse | SIN3B |
| ChEA3 2022 | CBX2 27304074 Chip-Seq ESCs Mouse | CBX2 |
| ChEA3 2022 | SUZ12 20075857 ChIP-Seq MESCs Mouse | SUZ12 |
| Reactome 2022 | RUNX1 Regulates Expression Of Components Of Tight Junctions R-HSA-8935964 | RUNX1 regulation of tight junctions |
| Reactome 2022 | TNFR1-mediated Ceramide Production  R-HSA-5626978 | TNFR1-mediated ceramide production |
| Reactome 2022 | Signaling By NTRK1 (TRKA) R-HSA-187037 | NTRK1 signaling |
| Reactome 2022 | Nuclear Events (Kinase And Transcription Factor Activation) R-HSA-198725 | Kinase and TF activation |
| Reactome 2022 | NGF-stimulated Transcription R-HSA-9031628 | NGF-induced transcription |
| MGI Mammalian Phenotype Level 4 2021 | decreased body height MP:0001255 | decreased body height |
| MGI Mammalian Phenotype Level 4 2021 | abnormal nervous system physiology MP:0003633 | abnormal NS physiology |
| MGI Mammalian Phenotype Level 4 2021 | abnormal dorsal root ganglion morphology MP:0000961 | abnormal DRG morphology |
| MGI Mammalian Phenotype Level 4 2021 | abnormal miniature excitatory postsynaptic currents MP:0004753 | abnormal mEPSCs |
| MGI Mammalian Phenotype Level 4 2021 | abnormal proprioceptive neuron morphology MP:0004297 | abnormal PN morphology |
